# Characterization of Fiber-Type Composition and Phosphoproteins of Fast- and Slow-Growing Broilers

**DOI:** 10.3390/ani16091311

**Published:** 2026-04-24

**Authors:** Yi Li, Weiran Huo, Kaiqi Weng, Jinlu Liu, Yingjie Gu, Yuchun Cai, Yang Zhang, Yu Zhang, Xuming Hu, Guohong Chen, Qi Xu

**Affiliations:** 1 Key Laboratory of Animal Genetics and Breeding and Molecular Design of Jiangsu Province, College of Animal Science and Technology, Yangzhou University, Yangzhou 225009, China; liyi@yzu.edu.cn (Y.L.); 13938700441@163.com (W.H.); 18705279093@163.com (K.W.); ljl15949082457@163.com (J.L.); 19905184823@163.com (Y.G.); mx120250902@stu.yzu.edu.cn (Y.C.); zyang@yzu.edu.cn (Y.Z.); yuzhang@yzu.edu.cn (Y.Z.); hxm@yzu.edu.cn (X.H.); 2 Molecular and Genetic Laboratory, College of Life Science, Henan Normal University, Xinxiang 453007, China

**Keywords:** chicken, skeletal muscle, muscle fiber types, phosphoproteomic, protein phosphorylation

## Abstract

Muscle fibers exhibit high plasticity: both the fast-twitch fiber type and slow-twitch fiber type can mutually transform under the regulation of phosphorylation. In this study, we characterized the muscle fiber profiles and phosphoproteomes of the *m. extensor digitorum longus* (EDL) and *m. soleus* (SOL) in slow-growing Xueshan chickens and fast-growing Ross 308 broilers. Fiber-type distribution was quantified by immunohistochemistry and RT-qPCR of *MYH7B*, *MYH1A* and *MYH1B*. TMT-based phosphoproteomics, combined with bioinformatic analysis, was used to identify differentially expressed phosphopeptides (DEPs) in two comparisons: Ross 308 SOL vs. Ross 308 EDL, and Xueshan EDL vs. Ross 308 EDL. The proportion of type I (slow-twitch) fibers in EDL was significantly higher in Xueshan chickens than in Ross 308 broilers (15.53% vs. 6.14%, *p* < 0.05), with no significant differences in fiber distribution or diameter between the SOL and EDL in Xueshan chickens (*p* > 0.05). A total of 3226 phosphopeptides corresponding to 1762 phosphoproteins were identified, with serine as the most abundant phosphorylated amino acid (73.92%). PDHA1, PHKB and PGAM1 were identified as the key DEPs common to both comparison groups. Bioinformatic analyses revealed that reversible site-specific phosphorylation regulates avian muscle fiber-type transformation mainly via the glycolysis/gluconeogenesis pathway.

## Simple Summary

Chicken meat quality is largely determined by the types of muscle fibers present—either “*fast-twitch*” fibers for quick movement or “*slow-twitch*” fibers for endurance. Here, we compared two different chicken breeds: the fast-growing Ross 308 broiler and the slow-growing Xueshan chicken, focusing on two key leg muscles. We discovered that the Xueshan chicken naturally possesses a higher proportion of slow-twitch fibers in certain muscles compared to Ross 308 broiler. In further research, we found that protein phosphorylation—a cellular molecular “switch”—regulates chicken muscle fiber type by controlling muscle energy metabolism pathways. Several core proteins were also identified, whose phosphorylation directly affects the shift between slow and fast fiber metabolism. Our findings help explain why different chicken breeds have different meat textures and provide a foundation for future farming practices to improve poultry growth and meat quality by understanding these natural molecular switches.

## 1. Introduction

Chicken meat is a globally essential source of high-quality protein, with its sensory attributes—such as tenderness, color, and water-holding capacity—fundamentally determined by the composition of skeletal muscle fiber types [1,2,3]. These fibers are categorized into two distinct phenotypes: type I (*slow-twitch*) fibers are characterized by a high mitochondrial density and elevated myoglobin content; they rely predominantly on aerobic oxidative phosphorylation for sustained energy production, which confers significant fatigue resistance. In contrast, type II (*fast-twitch*) fibers possess higher concentrations of glycogen and phosphocreatine, utilizing anaerobic glycolysis as the principal energy source to facilitate rapid and powerful contractions. These functional disparities are driven by the differential expression of myosin heavy-chain (MyHC) isoforms and the varying activities of key metabolic enzymes, such as succinate dehydrogenase (SDH) in oxidative fibers and lactate dehydrogenase (LDH) in glycolytic fibers. Crucially, the ratio of these fiber types dictates the contractile properties and post-mortem metabolic rate of the carcass. Research has indicated that muscles with a higher proportion of type II fibers often undergo rapid post-mortem glycolysis, leading to a faster pH decline, paler surface color, and increased drip loss [4,5]. Therefore, understanding the biochemical regulation of fiber-type proportions—specifically through molecular mechanisms like protein phosphorylation—represents a critical strategy for optimizing meat quality in the poultry industry.

Although muscle fiber number is fixed at birth, muscle fibers exhibit high plasticity: the fast-twitch fiber type and slow-twitch fiber type can mutually transform, as they are capable of responding to external stimuli by changing their phenotypic profile without changing their genotype [6,7]. In the process, protein phosphorylation/dephosphorylation seems to play a major role. Reversible phosphorylation/dephosphorylation of proteins is the best-known post-translational modification of proteins, playing a crucial role in protein structure, function, and activity regulation in muscle contraction and glycolysis, which may alter meat quality traits [8,9,10,11]. Previous work on fast and slow skeletal muscles has revealed that increased phosphorylation of the myosin light chain is associated with a slow-to-fast transition, regardless of whether hypertrophy or atrophy develops in rat soleus muscle [12]. Recent work on rabbit muscle revealed that myosin regulatory light chain phosphorylation at Ser17 can regulate muscle contraction and thus affect actomyosin dissociation and meat quality [11]. However, the phosphoproteins in slow and fast skeletal muscles and manipulation of fiber-type composition in individual muscles via phosphorylation of proteins have not been investigated in chicken.

Fast-growing commercial white-feathered broilers and slow-growing native yellow-feathered chickens, alongside spent laying hens, form the backbone of the Chinese modern poultry meat industry. Among these, the Xueshan chicken is an important locally developed and bred meat-type breed with a moderate growth rate. By contrast, the Ross 308 broiler represents a globally mainstream commercial line intensively selected for accelerated growth rate, elevated feed conversion efficiency and increased meat yield. In the present investigation, these two genetically divergent breeds were used to compare muscle fiber-type profiles in the EDL and soleus from legs. Additionally, comprehensive phosphoproteomic profiling was conducted to elucidate how site-specific protein phosphorylation modulates muscle fiber-type composition, thereby providing a comprehensive phosphoproteomic landscape of avian muscle fiber types and offering a molecular basis for future studies on muscle metabolism and meat quality improvement in poultry.

## 2. Materials and Methods

### 2.1. Ethical Approval

The use of animals was approved by the Institutional Animal Care and Use Committee of the School of Animal Science and Technology, Yangzhou University (Permit Number: YZUDWSY201903621, Permit Number: 10 March 2019).

### 2.2. Animal Slaughter and Sample Collection

**Figure 1:**
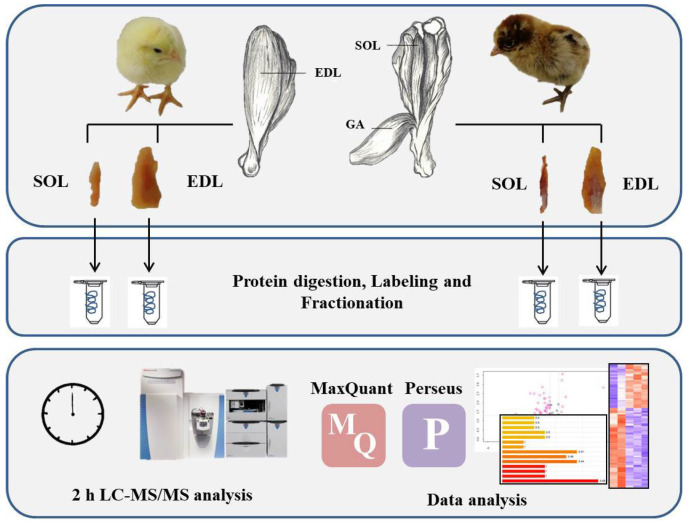
Sampling locations and experimental workflow. The diagram outlines the muscle sampling sites and the proteomic analysis process for Ross 308 broilers and Xueshan chickens. SOL, *m. soleus*; EDL, *m. extensor digitorum longus*; LC-MS/MS, liquid chromatography–tandem mass spectrometry; iTRAQ, isobaric tags for relative and absolute quantitation; MQ, MaxQuant; P, Perseus.

The experimental approach, including sample preparation, iTRAQ-based relative quantification, LC-MS/MS analysis, and data analysis, is illustrated in Figure 1. Fifteen one-day-old female hatchlings of each breed (Ross 308 broilers and Xueshan chickens) were obtained from LiHua Farming Co., Jiangsu, China. The birds were anesthetized with sodium pentobarbital, and killed by neck cut. The entire SOL and EDL were excised. For histological analysis, the mid-belly region was selected to ensure consistency. For phosphoproteomics, the whole muscle unit was utilized after removing the surrounding connective tissue to obtain adequate protein for analysis. Part of the samples (0.5 × 0.5 × 1.0 cm^3^) were cut and stored in 4% paraformaldehyde. Additionally, a subset of samples was wrapped in plastic film and chilled at 4 °C to maintain tissue stability. For protein extraction and subsequent phosphoproteomic analysis, samples were harvested at 24 h post-mortem and immediately snap-frozen in liquid nitrogen.

### 2.3. Immunohistochemistry Analysis

Serial sections with a thickness of 5 μm were prepared from paraffin-embedded muscle samples. For immunohistochemistry staining, sections were first deparaffinized in xylene and rehydrated through a graded series of ethanol [13]. In brief, tissue slides were sequentially subjected to deparaffinization in xylene (three cycles of 15 min each) and gradient rehydration in anhydrous, 95% and 80% ethanol (two cycles of 5 min each), followed by incubation in 3% methanol–hydrogen peroxide solution for 10 min to quench endogenous peroxidase activity. Slides were then probed overnight at 4 °C with primary antibodies targeting fast myosin skeletal heavy chain (MYH1A, 1:1200, ab51263, Abcam, Shanghai, China) or slow myosin skeletal heavy chain (MYH7B, 1:4000, ab11083, Abcam). Following three washes with PBS (5 min each), sections were incubated with rabbit anti-mouse IgG secondary antibody (1:5000, ab6728, Abcam) for 1 h. The biological signals were visualized using a 3,3′-diaminobenzidine DAB peroxidase substrate kit and then counterstained with hematoxylin. For each muscle sample, approximately 200 intact muscle fibers, free from tissue disruption or freezing artifacts, were randomly selected for analysis. All analyses were conducted using samples obtained from six broilers within each experimental group. The cross-sectional area (CSA, μm^2^), diameter (μm) and proportions (area %) of muscle fibers were quantified and calculated using Image-Pro Plus or ImageJ software 2.16 [14].

### 2.4. Purification of Total RNA and Relative mRNA Quantification

Total RNA was isolated from each muscle sample using the TRIzol reagent method. The concentration and purity of the extracted RNA were determined by measuring the optical density (OD) at 260 and 280 nm using a spectrophotometer (Thermo Scientific, Waltham, MA, USA). Only high-purity RNA samples were utilized for subsequent experiments, while the remaining samples were archived at −80 °C. Genomic DNA (gDNA) removal and first-strand cDNA synthesis were performed using the FastKing One-Step gDNA Removal and cDNA Synthesis Kit (Tiangen, Beijing, China). The reverse transcription program was set at 42 °C for 15 min, followed by 15 s at 95 °C for 3 min. The resulting cDNA was stored at −20 °C for further analysis.

Specific primers for real-time quantitative PCR (RT-qPCR) were designed via Premier 5.0 software and synthesized by Tsingke Biotech Co., Ltd. (Beijing, China) (Table S1). RT-qPCR was conducted using a commercial kit (Vazyme Biotech Co., Ltd., Nanjing, China) according to a three-step protocol [15]. The thermal cycling conditions were as follows: initial denaturation at 95 °C for 2 min, followed by 40 cycles of 95 °C for 10 s and 60 °C for 15 s. The melting curve analysis was performed at 95 °C for 15 s, 60 °C for 60 s, and 95 °C for 15 s. Relative mRNA expression levels were calculated using the 2^−ΔΔCt^ method [16].

### 2.5. Phosphoproteomic Analysis

#### 2.5.1. Protein Extraction, Digestion and Tandem Mass Tag (TMT) Labeling

Protein extraction was performed as previously described [17]. The filtrate was collected and stored at −80 °C. Protein was digested according to a procedure from the literature [18]. Peptide content was determined using a ultraviolet spectrophotometer at 280 nm [19]. Finally, 100 μg of peptide was labeled using TMT 6 plex Mass Tag Labeling Kits and Reagents (Thermo Fisher Scientific, Waltham, MA, USA) according to the manufacturer’s instructions.

#### 2.5.2. Phosphopeptide Enrichment and LC-MS/MS Analysis

The enrichment of phosphorylated peptides was performed according to the methodology described by Thingholm [20]. Briefly, phosphopeptides were selectively isolated from the complex peptide mixture and subsequently concentrated using a reverse-phase trap column (Acclaim PepMap100, 100 μm × 2 cm, nanoViper C18; Thermo Scientific). High-resolution chromatographic separation was achieved on a C18-reversed phase analytical column (Easy Column, 25 cm long, 75 μm inner diameter, 3 μm resin; Thermo Scientific). The resulting eluates were analyzed via a Q Exactive mass spectrometer (Thermo Scientific) coupled with an Easy nLC system (Proxeon Biosystems, now Thermo Fisher Scientific). The detailed MS acquisition parameters and liquid chromatography gradients followed the procedures previously established [21].

#### 2.5.3. Bioinformatic Analysis

MS/MS raw spectra were processed by Proteome Discoverer 1.4 (Thermo Fisher Scientific, San Jose, CA, USA) and searched against the Uniprot *Gallus_gallus* database (accessed on 14 October 2019) using the Mascot 2.2 engine [22]. To ensure the reliability of identification, a decoy database search was simultaneously performed. The quantitative assessment of phosphoproteins followed the methodology established in our previous study [21]. Relevant technical parameters are detailed in Table S2. For functional interpretation, the identified proteins were subjected to systematic bioinformatic analyses, including Gene Ontology (GO) enrichment, Kyoto Encyclopedia of Genes and Genomes (KEGG) pathway mapping, and hierarchical cluster analysis.

### 2.6. Statistical Analysis

Data were presented as means ± standard error of the mean (SEM) and analyzed using the SPSS package (version 17.0, SPSS Inc., Chicago, IL, USA). One-way ANOVA followed by Duncan’s multiple range test was used to compare RT-qPCR data between groups. A two-way ANOVA was performed to test the main effects of breed and muscle type, as well as their interaction. Differences were considered statistically significant when *p* < 0.05, and highly significant at *p* < 0.01.

## 3. Results

### 3.1. Muscle Fiber Type of Chicken Skeletal Muscles

**Figure 2:**
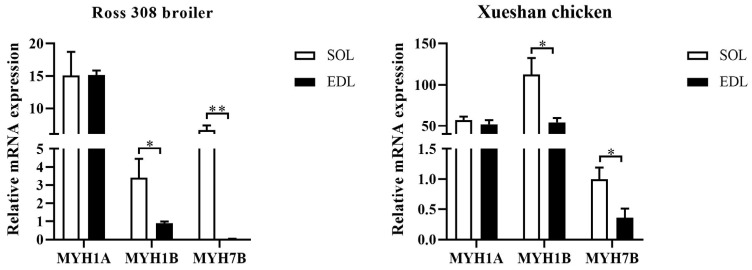
Relative mRNA expression of myosin heavy0chain (MyHC) isoforms in muscle from chickens of two breeds. *MYH1A*, type IIb, fast-twitch; *MYH1B*, type IIa, fast-twitch; *MYH7B*, type I, slow-twitch. Vertical bars represent mean ± SE (n = 6). Asterisks denote significant differences between SOL and EDL (* *p* < 0.05, ** *p* < 0.01).

The results of this study are shown in Figure 2. There was little expression of *MYH7B*, and no significant difference was observed in muscular tissues from Xueshan chickens (*p* > 0.05). In addition, the SOL exhibited higher expression of *MYH7B* than did the EDL in Ross 308 broilers (*p* < 0.05), and the relative expression of *MYH7B* was lower than that of *MYH1A*.

**Figure 3:**
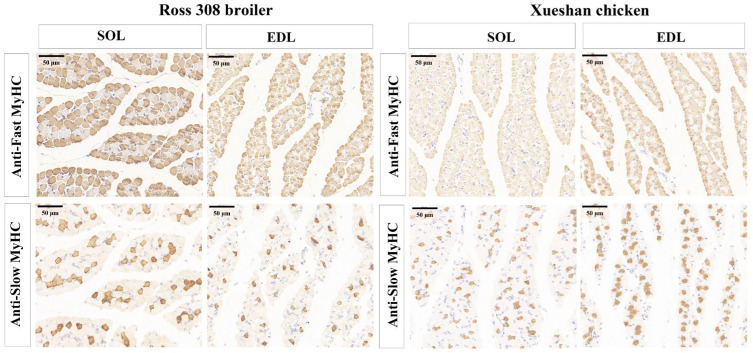
Immunohistochemical analyses of muscle fiber in Ross 308 broilers and Xueshan chickens (Scale bar = 50 μm).

To confirm the distribution of MHCs at the protein level, immunohistochemistry was performed. Based on the data shown in Figure 3, two MHC-based fiber types, type I (slow-twitch) and type II (fast-twitch), were detected in four chicken muscles. Representative characteristics such as fiber diameter, fiber area composition and CSA are presented in Table 1. Compared with Ross 308 broilers, Xueshan chickens exhibited significant differences in the diameter, CSA, and area percentage of type I and type II muscle fibers in both the SOL and EDL (*p* < 0.05 or *p* < 0.01). Breed exerted a significant or highly significant main effect on all fiber characteristics (*p* < 0.05 or *p* < 0.01), while muscle type showed a significant effect only on fiber diameter and CSA (*p* < 0.01), with no effect on fiber area percentage. A significant breed–muscle type interaction was also detected for all indicators. These results strongly suggest that breed is a key factor driving muscle fiber development in broilers.

In Xueshan chickens, SOL muscle and EDL muscle had no significant difference in fiber diameter between the fiber types (*p* > 0.05). Higher fiber diameter was observed in type II than in type I in SOL muscle from Ross 308 broilers, whereas EDL muscle had an opposite trend in fiber diameter between the fiber types. A comparison of the fiber compositions among the muscles revealed that the SOL and EDL muscles were predominantly composed of fast-twitch muscle fibers, while the percentage of slow-twitch fiber in the EDL was higher in Xueshan chickens (15.52%) when compared to Ross 308 chickens (6.14%). Similarly, the percentage of slow-twitch fibers in the SOL was higher (13.51%) when compared to the EDL in Ross 308 broilers (6.14%), while no difference in the percentage of fibers between the SOL and EDL from Xueshan chickens was observed. Taken together, these data demonstrated that Xueshan chickens had a higher percentage of slow-twitch fibers in the EDL and that the percentage of slow-twitch fibers in the SOL was higher when compared to the EDL in Ross 308 broilers.

### 3.2. Phosphoprotein Identification and Motif Analysis

To examine the difference in phosphoproteins between slow and fast muscle fiber types, iTRAQ labeling combined with LC–MS/MS was used. A total of 3226 phosphopeptides were identified, including 1762 phosphoproteins. Supplementary Figure S1 shows that serine was the most common phosphorylated amino acid (73.92%), followed by threonine (16.12%) and tyrosine (9.96%). Thirty-four putative phosphorylation motifs were identified after analysis through the Motif-X software (v2.0), as well as three types of motifs, including the proline-directed kinase substrate motif (pSP, pTP, and RXXpSP) and basophilic motif (RXXpSP) (Supplementary Figure S2).

**Figure 4:**
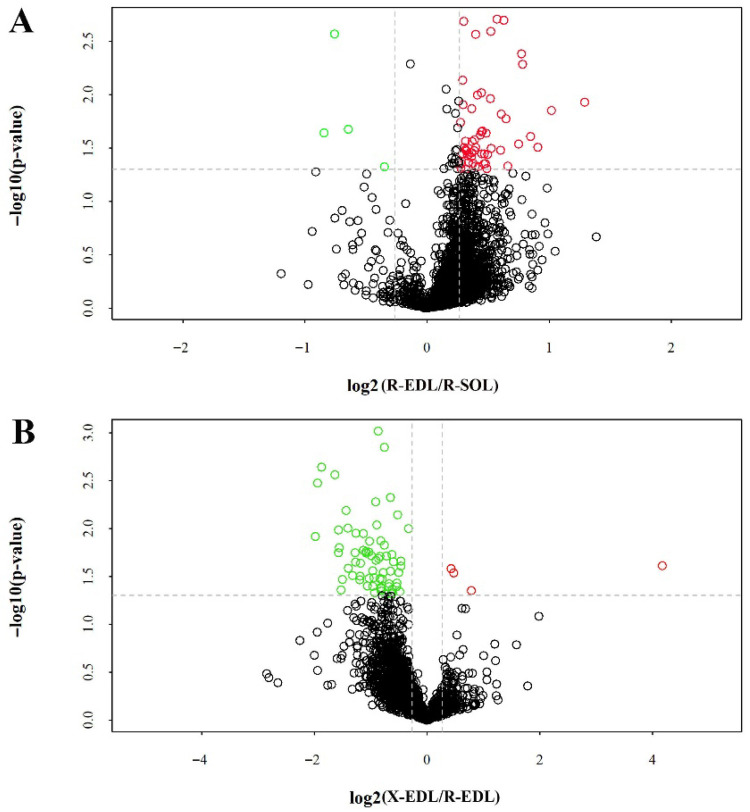
Differentially regulated phosphopeptides were identified by plotting the −log10 *p*-values and log2 fold changes in reported intensities. (**A**) EDL vs. SOL in Ross 308 broilers. (**B**) EDL in Xueshan chickens vs. EDL in Ross 308 broilers. Fold change set to >1.2 (red) or <0.83 (green) with *p* < 0.05.

We further screened the phosphopeptides by setting a fold change threshold of >1.2 or <0.83 and a *t*-test *p*-value of <0.05. As a result (Figure 4, Supplementary Table S3), we identified 59 up-regulated phosphopeptides (corresponding to 54 phosphoproteins) and four down-regulated phosphopeptides (corresponding to four phosphoproteins) in EDL muscle compared to SOL muscle from Ross 308 broilers. In addition, five up-regulated phosphopeptides (corresponding to four phosphoproteins) and 72 down-regulated phosphopeptides (corresponding to 66 phosphoproteins) were identified in EDL muscle from Xueshan chickens compared to EDL muscle from Ross 308 broilers.

### 3.3. Hierarchical Clustering Analysis

**Figure 5:**
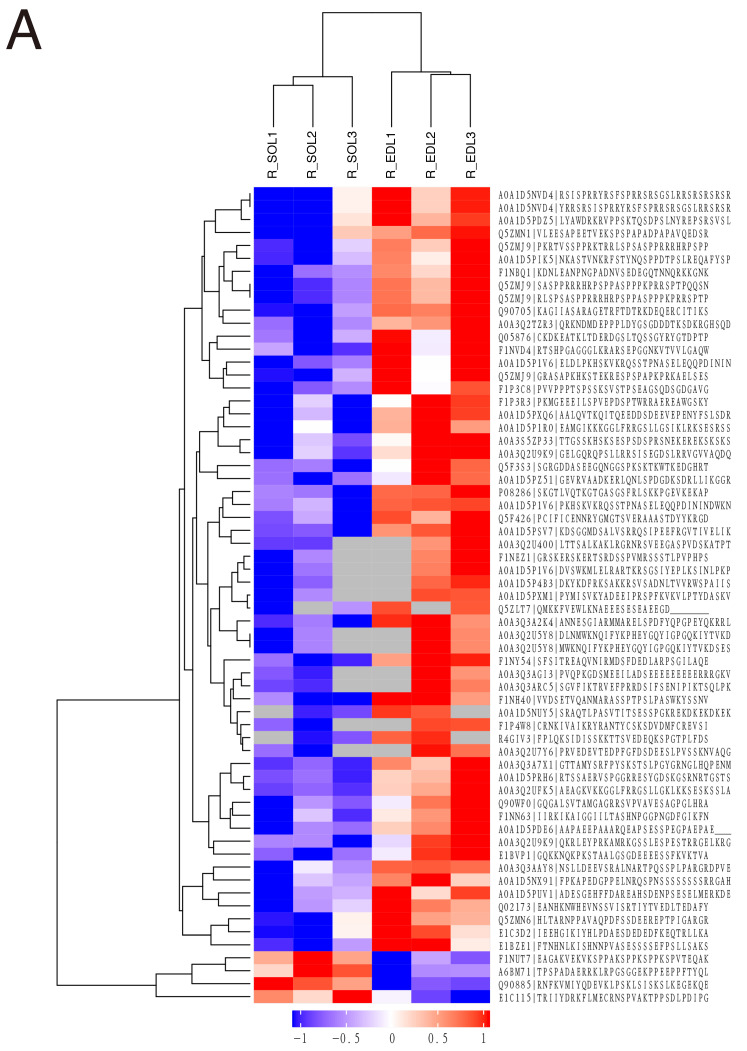

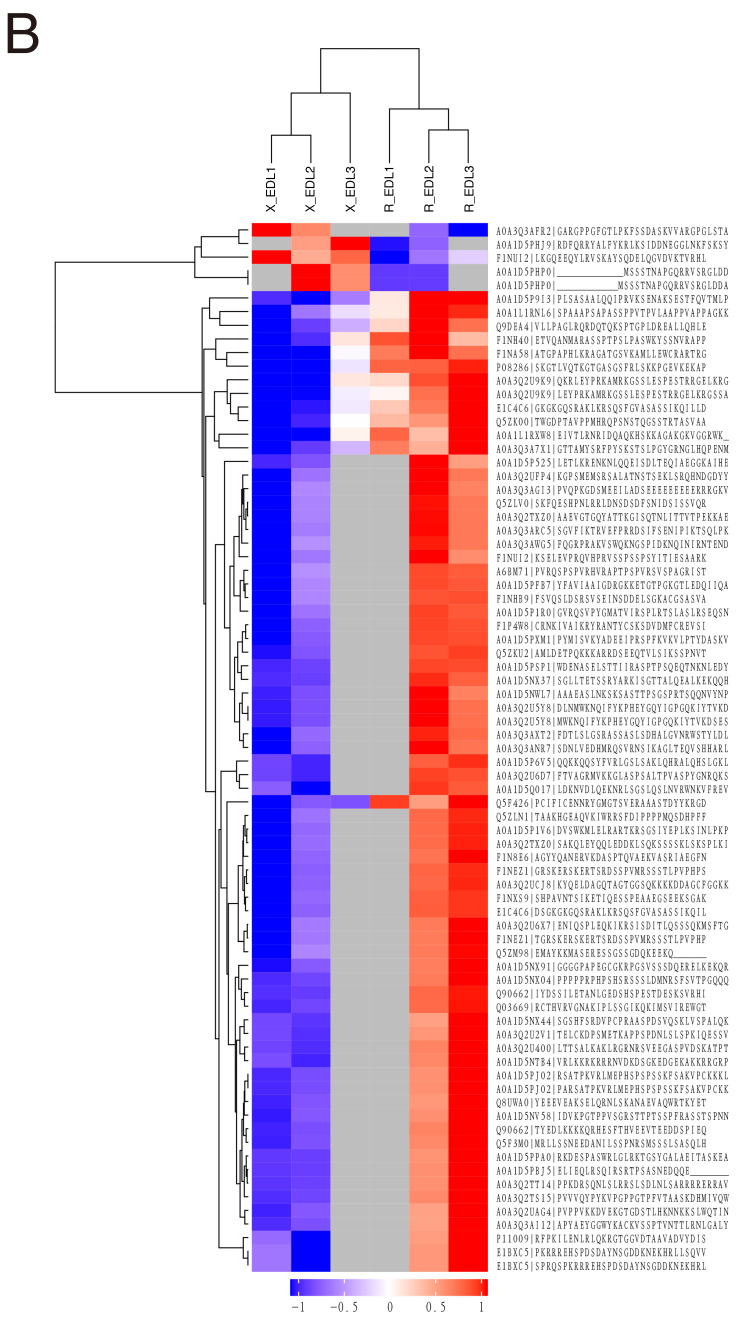
Hierarchical clustering of different phosphopeptides. (**A**) EDL vs. SOL in Ross 308 broilers. (**B**) EDL in Xueshan chickens vs. EDL in Ross 308 broilers. A redder hue indicates a higher level of phosphorylation and a bluer hue indicates a lower level of phosphorylation in phosphopeptides.

Subsequently, the phosphopeptides that displayed significant differences in phosphorylation levels were subjected to hierarchical clustering analysis. As shown in Figure 5, the color distributions of the three biological replicates were similar in each group and the two groups represented characteristic patterns of phosphorylation, which indicated an obvious specificity and reproducibility. Interestingly, high levels of phosphorylation in SOL muscles were often associated with low levels of phosphorylation in EDL muscles of Ross 308 broilers, and vice versa.

### 3.4. GO Functional Classification of Differentially Phosphorylated Proteins

**Figure 6:**
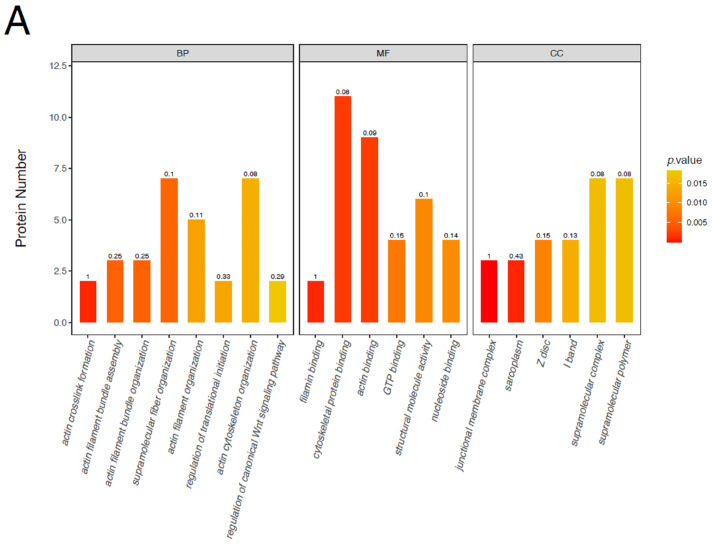

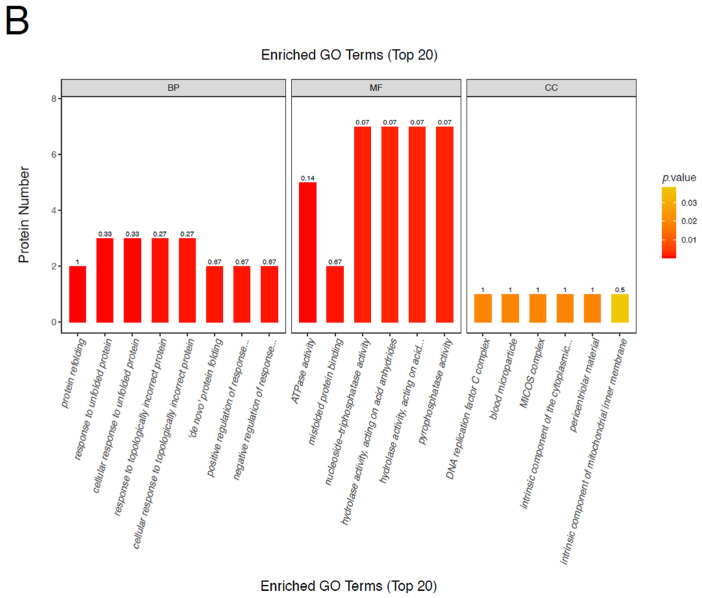
GO terms for enrichment of differentially phosphorylated proteins. (**A**) EDL vs. SOL in Ross 308 broilers. (**B**) EDL in Xueshan chickens vs. EDL in Ross 308 broilers. The abscissa in the (**A**,**B**) indicates the enriched GO function. BP, MF and CC represent biological process, molecular function and cellular component respectively.

To determine the functional classification of all differentially phosphorylated proteins, these differentially phosphorylated proteins were analyzed according to their molecular function (MF), biological process (BP), and cellular component (CC). Figure 6 shows the distribution of the number of differentially phosphorylated proteins implicated in MF, BP, and CC. Most differentially expressed phosphoproteins between the EDL and SOL in the Ross 308 broiler group were involved in actin crosslink formation, actin filament bundle assembly, actin filament bundle organization, supramolecular fiber organization, filamin binding, cytoskeletal protein binding, actin binding, junctional membrane complex and sarcoplasm. Similarly, the differentially expressed phosphoproteins between the EDL in Xushan chickens and the EDL in Ross 308 broilers were mainly distributed in the actin binding, nucleoside-triphosphatase activity, hydrolase activity acting on acid anhydrides, hydrophosphatase activity, cytoskeletal protein binding, cytoskeleton, cytoskeletal part, and cytoskeleton organization.

### 3.5. KEGG Pathway Analysis of Differentially Phosphorylated Proteins

**Figure 7:**
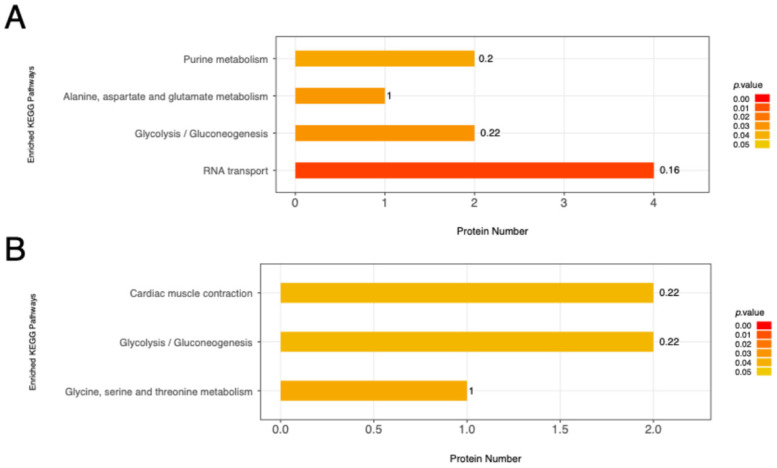
KEGG pathway enrichment of differentially phosphorylated proteins. (**A**) EDL vs. SOL in Ross 308 broilers. (**B**) EDL in Xueshan chickens vs. EDL in Ross 308 broilers. The numbers beside the bars are rich factors, which indicate the ratio of proteins corresponding to phosphopeptides that differ significantly in phosphorylation levels to all identified proteins.

To obtain some information about the mechanism regulating fiber type, we annotated the differentially phosphorylated proteins through enrichment analysis in KEGG (Figure 7). Based on KEGG analysis, we identified that there were four significantly enriched pathways, namely RNA transport, glycolysis/gluconeogenesis, purine metabolism and alanine, aspartate and glutamate metabolism between the EDL and SOL in the Ross 308 broiler group, and three pathways (glycine, serine and threonine metabolism, glycolysis/gluconeogenesis and cardiac muscle contraction) were assigned to differentially phosphorylated proteins between the EDL in Xushan chickens and the EDL in Ross 308 broilers. Together, functional enrichment analysis of differential phosphoproteins revealed that phosphoproteins principally focus on these glycolysis maps, particularly glycolysis/gluconeogenesis.

### 3.6. Functional Annotation Analysis of Key Differentially Regulated Phosphoproteins

Functional annotation of the key regulated phosphoproteins related to the muscle types is listed, with the corresponding fold changes and *p* values, in Table 2. EDL muscle compared to SOL muscle in the Ross 308 broiler group exhibited five key phosphoproteins—four phosphoproteins were up-regulated and one was down-regulated—while EDL muscle from Xueshan chickens compared to EDL muscle from the Ross 308 broiler group exhibited ten key down-regulated phosphoproteins. Among the key differentially regulated phosphoproteins, pyruvate dehydrogenase E1 component subunit alpha (PDHA1), phosphorylase b kinase regulatory subunit (PHKB), and connectin (Fragment, TTN) were found in two groups. Uncharacterized proteins, including ABLIM2 and PGM1, were found in EDL muscle compared to SOL muscle in the Ross 308 broiler group, and phosphoglycerate mutase 1 (PGAM1), creatine kinase S-type, mitochondrial (CKMT2), uncharacterized protein (MYBPC1), myosin-1B (MYH1B), troponin T, fast skeletal muscle isoforms (TNNT3), sarcoplasmic/endoplasmic reticulum calcium ATPase 2 (ATP2A2) and myosin heavy chain (SSMHC) were found in EDL muscle from the Xueshan chicken group compared to EDL muscle from the Ross 308 broiler group. Taken together, these key phosphoproteins were mainly involved in muscle contraction, glycolysis and oxidative phosphorylation.

## 4. Discussion

Skeletal muscles contain slow-twitch (type I) and fast-twitch (type II) muscle fibers, which are responsible for different contractile properties and metabolic functions in most animals [1,2]. This composition pattern varies greatly between mammals and avians. In mammals, the EDL is mainly composed of type II fibers, whereas the SOL is a muscle predominantly or entirely composed of type I fibers [23,24,25]. By contrast, in this study, the SOL and EDL muscles were predominantly composed of fast-twitch muscle fibers in Xueshan chickens (86.67% and 84.47%, respectively) and Ross 308 broilers (86.49% and 93.86%, respectively). Further analysis revealed that the proportion of fast-twitch muscle fibers in EDL muscle of Ross 308 broilers was significantly higher than that of Xueshan chickens. This difference may be closely related to the breeding directions of the two chicken breeds: as a commercially selected fast-growing broiler breed subjected to intensive selection, Ross 308 broilers have breeding objectives focused on rapid growth and high meat yield [26]. Fast-twitch muscle fibers are characterized by fast contraction speed and large fiber diameter, enabling the rapid accumulation of sarcoplasmic proteins, which is consistent with its breeding demand for high meat yield. We also found that the percentage of slow-twitch fibers in the EDL was higher in Xueshan chickens (15.53%) when compared to Ross 308 broilers (6.14%). Similarly, the percentage of slow-twitch fibers in the SOL was higher (13.51%) compared to that in the EDL in Ross 308 broilers (6.14%), while no difference in the percentage of fibers between the SOL and EDL was observed Xueshan chickens. However, the molecular mechanism regulating fiber-type composition in chicken still remains a challenge.

The phosphorylation of the proteins has been recognized to play a crucial role in glycolysis and muscle contraction, directly or indirectly regulating muscle fiber-type composition [11,12,27,28]. In the present work, phosphoproteomic analysis identified several key regulated phosphoproteins related to muscle types, including MYBPC1, MYH1B, TNNT3, SSMHC and others. In avians, MYH1B (known as MYH3 in mice) can regulate muscle fiber-type conversion in skeletal muscle [29]. MYH3 can promote slow-twitch muscle fibers but has no significant effect on fast-twitch muscle fibers in mice [30]. Another study reported that MYH1B could induce the slow-twitch muscle phenotype in chicken, consistent with the function of MYH3 in mice [31]. Similarly, TNNT3 is specifically expressed in fast-twitch skeletal muscle fibers and plays a key role in calcium-dependent regulation of thin-filament functions [32]. MYBPC1 encodes myosin-binding protein-C slow (sMyBP-C), a regulatory protein in skeletal muscles that can be phosphorylated by PKA and PKC [33]. Previous studies have also shown that phosphorylation of myosin heavy-chain proteins may be related to muscle protein stability [34]. In fish, up-regulated phosphorylation of this major myosin heavy chain may protect muscle fibers from proteolytic degradation [35]. However, few reports have addressed the phosphorylation of MYH1B, MYBPC1,TNNT3, SSMHC and myosin heavy chain in poultry skeletal muscle. We found that MYH1B Ser1515, MYBPC1 Ser891, TNNT3 Ser206 and SSMHC Ser1370 were differentially phosphorylated in the EDL muscle of Xueshan chickens compared with Ross 308 broilers. Because these proteins are closely related to myofibrillar structure and contractile regulation, phosphorylation at these sites may be associated with muscle fiber-type transition rather than merely reflecting differences in expression levels. Our study provides novel insights by identifying these specific phosphorylation sites associated with muscle fiber-type transition, indicating candidate regulatory sites for the modulation of leg muscle fiber-type composition in chickens.

KEGG pathway analysis in this study identified significant enrichment of glycolysis/gluconeogenesis, purine metabolism, and alanine, aspartate and glutamate metabolism. It is well known that different muscle fiber types rely on distinct metabolic programs to support contraction [28]. Specifically, glycolytic fibers can predominantly metabolize glucose, and oxidative fibers rely on energy produced by oxidative phosphorylation [36,37]. Our list of putative regulatory phosphosites confirmed the fiber-type-specific phosphorylation level of phosphoproteins associated with glycolysis and oxidative phosphorylation. Among the glycolytic-related phosphoproteins, PGM1, PGAM1 and PDHA1 are the key players involved in glycolytic flux and ATP generation for muscle contraction [38,39,40]. In this study, these three proteins were identified to be the glycolytic rate-related phosphoproteins, which were detected to be phosphorylated at Ser117 (PGM1), Ser118 (PGAM1), and Ser329 (PDHA1), respectively. In addition, we examined PHKB, a regulatory subunit of glycogen phosphorylase kinase. In Ross 308 broilers, PHKB was phosphorylated at Ser29, Ser710 and Thr706 in the EDL compared to the SOL. However, only Ser17 was identified as a key site regulating PHKB phosphorylation in the EDL of Xueshan chickens compared with that of Ross 308 broilers. Different phosphorylation sites within the same protein may exert distinct regulatory effects and may not necessarily lead to the same downstream outcomes. In the present study, several metabolism-related proteins, including PGM1, PGAM1, PDHA1, and PHKB, showed relatively higher phosphorylation levels in the fast-twitch phenotype than in the slow-twitch phenotype. This pattern suggests that reversible phosphorylation of metabolic enzymes may be involved in the maintenance or formation of glycolytic fast-twitch fibers in chicken skeletal muscle. However, whether these specific phosphosites directly alter enzyme activity, metabolic flux, or downstream signaling pathways requires further functional validation.

## 5. Conclusions

This study demonstrates that site-specific phosphorylation is a central regulator of muscle fiber plasticity in chickens. Slow-growing Xueshan chickens display a significantly higher proportion of slow-twitch fibers in the EDL muscle than fast-growing Ross 308 broilers. Phosphoproteomic profiling identified 1762 phosphoproteins, with serine as the predominant phosphorylation site. PDHA1, PHKB, and PGAM1 were core shared differentially phosphorylated proteins, indicating that reversible phosphorylation modulates fiber-type switching mainly through the glycolysis/gluconeogenesis pathway. These results provide a molecular basis for targeted manipulation of muscle fiber composition to improve poultry meat quality. Elucidating the kinase–phosphatase networks governing these key events will further refine strategies for optimizing muscle metabolism and meat quality in broilers.

## Figures and Tables

**Table 1:** Muscle fiber characteristics of SOL and EDL muscles in Ross 308 broilers and Xueshan chickens.

	Ross 308 Broiler		Xueshan Chicken		SEM	*p*-Value		
	SOL	EDL	SOL	EDL		Breed	Muscle Type	Breed × Muscle Type
Type II fiber, μm	13.94	7.78	8.72	10.43	0.15	<0.001	<0.001	<0.001
Type I fiber, μm	11.17	8.80	8.86	10.37	0.17	0.024	0.011	<0.001
Mean (I + II), μm	12.62	8.32	8.77	10.40	0.12	<0.001	<0.001	<0.001
Fiber cross-sectional area, μm^2^	206.65	117.76	92.66	140.07	7.77	<0.001	0.0081	<0.001
Type I fiber area percentage,%	13.51	6.14	13.33	15.52	1.44	0.016	0.970	0.023
Type II fiber area percentage, %	86.49	93.86	86.66	84.47	1.44	0.016	0.970	0.023

Note: Type I: *slow-twitch fiber*. Type II: *fast-twitch fiber*. SEM, standard error of the mean.

**Table 2:** Selection of fiber specific regulated phosphoproteins.

Accession	Protein/Gene Name	Sequence	Positions Within Proteins	Amino Acid	Fold Change	*p*-Value
EDL VS SOL in Ross 308 broiler						
Q5F426	Pyruvate dehydrogenase E1 component subunit alpha/PDHA1	PCIFICENNRYGMGTSVERAAASTDYYKRGD	239	S	2.445	0.011
A0A3Q3A7X1	Uncharacterized protein/ABLIM2	GTTAMYSRFPYSKSTSLPGYGRNGLHQPENM	512	S	2.023	0.014
A0A1D5P1V6	Phosphorylase b kinase regulatory subunit/PHKB	DVSWKMLELRARTKRSGSIYEPLKSINLPKP	29	S	1.437	0.031
A0A1D5P1V6	Phosphorylase b kinase regulatory subunit/PHKB	ELDLPKHSKVKRQSSTPNASELEQQPDININ	706	T	1.360	0.022
A0A1D5P1V6	Phosphorylase b kinase regulatory subunit/PHKB	PKHSKVKRQSSTPNASELEQQPDININDWKN	710	S	1.288	0.013
F1NN63	Uncharacterized protein/PGM1	IIRKIKAIGGIILTASHNPGGPNGDFGIKFN	117	S	1.298	0.044
A6BM71	Connectin (Fragment)/TTN	TPSPADAERRKLRPGSGGEKPPEEPPFTYQL	5333	S	0.556	0.022
EDL in Xueshan chicken VS EDL in Ross 308 broiler						
Q5ZLN1	Phosphoglycerate mutase 1/PGAM1	TAAKHGEAQVKIWRRSFDIPPPPMQSDHPFF	118	S	0.660	0.022
P11009	Creatine kinase S-type, mitochondrial/CKMT2	RFPKILENLRLQKRGTGGVDTAAVADVYDIS	356	T	0.654	0.046
A0A3Q3AWG5	Uncharacterized protein/MYBPC1	FQGRPRAKVSWQKNGSPIDKNQINIRNTEND	891	S	0.595	0.048
A6BM71	Connectin (Fragment)/TNN	PVRQSPSPVRHVRAPTPSPVRSVSPAGRIST	301	T	0.566	0.042
A0A1D5P525	Myosin-1B/MYH1B	LETLKRENKNLQQEISDLTEQIAEGGKAIHE	1515	S	0.487	0.017
A0A1D5P1V6	Phosphorylase b kinase regulatory subunit/PHKB	DVSWKMLELRARTKRSGSIYEPLKSINLPKP	29	S	0.472	0.017
A0A1L1RXW8	Troponin T, fast skeletal muscle isoforms/TNNT3	EIVTLRNRIDQAQKHSKKAGAKGKVGGRWK_	206	S	0.437	0.034
Q03669	Sarcoplasmic/endoplasmic reticulum calcium ATPase 2/ATP2A2	RCTHVRVGNAKIPLSSGIKQKIMSVIREWGT	538	S	0.369	0.006
Q8UWA0	Myosin heavy chain/SSMHC	YEEEVEAKSELQRNLSKANAEVAQWRTKYET	1370	S	0.352	0.033
Q5F426	Pyruvate dehydrogenase E1 component subunit alpha/PDHA1	PCIFICENNRYGMGTSVERAAASTDYYKRGD	239	S	0.272	0.002

## Data Availability

All data generated or analyzed during this study are included in this published paper.

## Supplementary Materials

The following supporting information can be downloaded at: https://www.mdpi.com/article/10.3390/ani16091311/s1, Figure S1: Phosphorylated amino acid; Figure S2: Phosphorylation-specific motifs using the MEME software (v5.5.9); Table S1: Primers for qRT-PCR of MyHC-related genes; Table S2: Set parameters and database used in quantitative phosphoproteomic analysis; Table S3: Number of differentially expressed phosphopeptides.
