# Supplementary material for: Characterization of Fiber-Type Composition and Phosphoproteins of Fast- and Slow-Growing Broilers
Source: Animals (Basel). 2026 Apr 24;16(9):1311. doi: 10.3390/ani16091311 (PMC13162959; doi:10.3390/ani16091311)
Supplement: Supplementary file 1 [file animals-16-01311-s001.zip › animals-4205140-Supplementary Table S1.pdf]

Table S1 Primers for qRT-PCR of MyHC-related genes.

Gene	GenBank accession	Primer sequence (5'→3')	Length (bp)	Product size (bp)	Annealing temperature (°C)
MYH7B	NM_204587.2	F: ACGGGCCTGATCAACCAAAA	20	114	60
		R: GGCCTTCTTGGCTTTCTCCT	20		
MYH1A	NM_001013396.1	F: GGTCAACAAGCTCCGAGTGA	20	92	60
		R: CAGGCCACTTTACTGCCTCA	20		
MYH1B	NM_204228.3	F: GGGAGACCTGAATGAAATGGAG	22	140	60
		R: CTTCCTGTGACCTGAGAGCATC	22		
18SrRNA	AF173612.1	F: GCGGCTTTGGTGACTCTA	18	194	60
		R: CTGCCTTCCTTGGATGTG	18		

MyHC, myosin heavy-chain. *MYH7B*, (type I) slow-twitch myosin heavy-chain; *MYH1A*, (type IIb) fast-twitch myosin heavy-chain; *MYH1B*, (type IIa) fast-twitch myosin heavy-chain.
