# Supplementary material for: Characterization of Fiber-Type Composition and Phosphoproteins of Fast- and Slow-Growing Broilers
Source: Animals (Basel). 2026 Apr 24;16(9):1311. doi: 10.3390/ani16091311 (PMC13162959; doi:10.3390/ani16091311)
Supplement: Supplementary file 1 [file animals-16-01311-s001.zip › animals-4205140-Supplementary Table S2.pdf]

Table S2. Set parameters and database used in quantitative proteomic analysis.

Item	Parameter/Methods/Software/Database
Enzyme	Trypsin
Max Missed Cleavages	2
Main Search	6 ppm
First Search	20 ppm
MS/MS Tolerance	±20 ppm
Fixed modifications	Carbamidomethyl (C), TMT 6 plex (N-term), TMT 6 plex (K)
Variable modifications	Oxidation (M), Acetyl (Protein N-term), Phospho (STY)
Database	uniprot_Anas_platyrhynchos_44952_20190909
Database pattern	Reverse
Include contaminants	True
Peptide FDR	⩽ 0.01
Site FDR	⩽ 0.01
Protein FDR	⩽ 0.01
Hierarchical clustering analysis	Cluster 3.0, http://bonsai.hgc.jp/~mdehoon/ software/cluster/software.htm Java Treeview software, http://jtreeview.sourceforge.net
Motif analysis	MEME, http://meme-suite.org/index.htm
GO annotation	Blast2GO, https://www.ncbi.nlm.nih.gov/protein/
KEGG annotation	KEGG database, http://geneontology.org/
GO enrichment and KEGG pathway enrichment analysis	Fisher' Exact Test
Protein–protein interaction	STRING datebase, https://string-db.org/
