# Supplementary material for: Characterization of Fiber-Type Composition and Phosphoproteins of Fast- and Slow-Growing Broilers
Source: Animals (Basel). 2026 Apr 24;16(9):1311. doi: 10.3390/ani16091311 (PMC13162959; doi:10.3390/ani16091311)
Supplement: Supplementary file 1 [file animals-16-01311-s001.zip › animals-4205140-Supplementary Table S3.pdf]

Table S3. Number of differentially expressed phosphopeptides.

Comparisons	Increased	Decreased	Total number of differentially expressed phosphopeptides	Total number of differentially expressed phosphoproteins
Ross 308 broiler SOL_vs_ Ross 308 broiler EDL	59	4	63	58
Xueshan chichen EDL_vs_ Ross 308 broiler EDL	5	72	77	66

M. extensor digitorum longus (EDL), and *m. soleus* (SOL).

Fold change set as >1.2 or <0.83 with $P < 0.05$.
