# Supplementary figures and images for: Characterization of Fiber-Type Composition and Phosphoproteins of Fast- and Slow-Growing Broilers
Source: Animals (Basel). 2026 Apr 24;16(9):1311. doi: 10.3390/ani16091311 (PMC13162959; doi:10.3390/ani16091311)

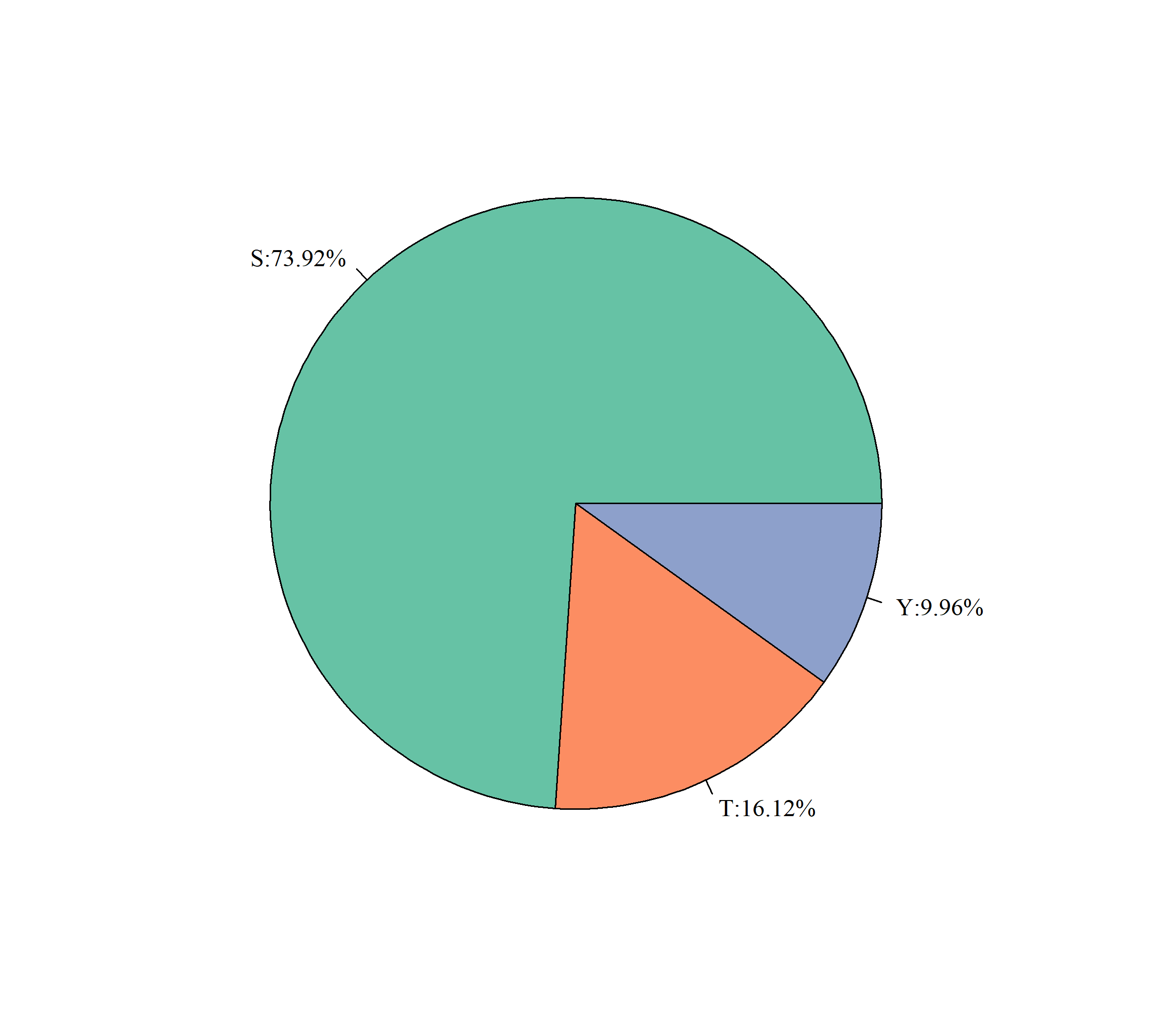

Supplement: Supplementary file 1 [file animals-16-01311-s001.zip › animals-4205140-Supplementary Figure S1.png]

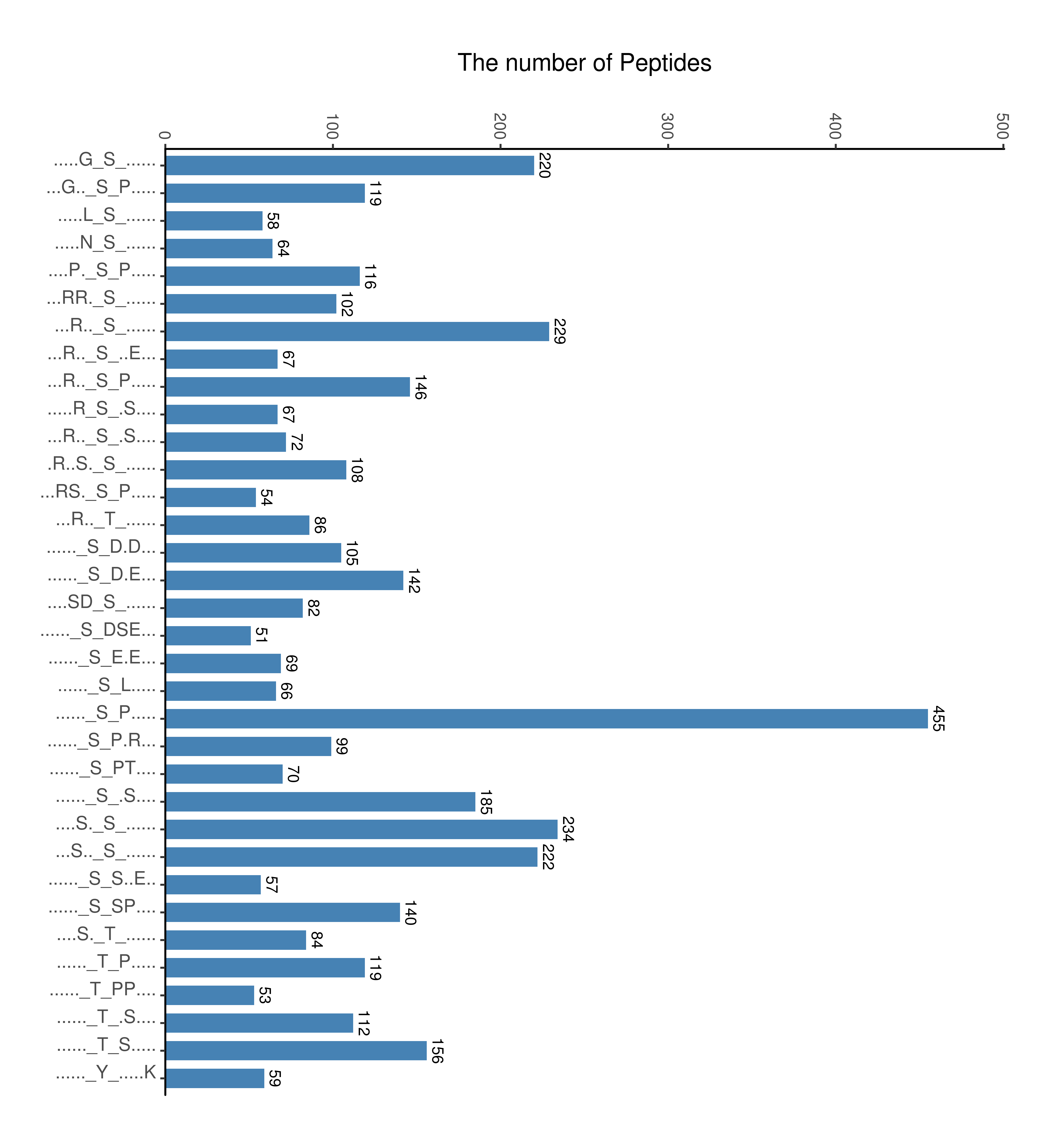

Supplement: Supplementary file 1 [file animals-16-01311-s001.zip › animals-4205140-Supplementary Figure S2.png]
